# Endothelial and Hemodynamic Function in a Large Animal Model in Relation to Different Extracorporeal Membrane Oxygenation Cannulation Strategies and Intra-Aortic Balloon Pumping

**DOI:** 10.3390/jcm12124038

**Published:** 2023-06-13

**Authors:** Stephen Gerfer, Ilija Djordjevic, Johanna Maier, Ana Movahed, Mara Elskamp, Elmar Kuhn, Oliver Liakopoulos, Thorsten Wahlers, Antje C. Deppe

**Affiliations:** 1 Department of Cardiothoracic Surgery, Heart Center, University Hospital of Cologne, University of Cologne, 50924 Cologne, Germany; 2 Division of Thoracic and Cardiovascular Surgery, HELIOS Klinikum Siegburg, 53721 Siegburg, Germany; 3 Department of Cardiac Surgery, Kerckhoff-Clinic Bad Nauheim, Campus Kerckhoff, University of Giessen, 61231 Bad Nauheim, Germany

**Keywords:** mechanical circulatory support, extracorporeal life support, ECLS, extracorporeal membrane oxygenation, ECMO, intra-aortic balloon pump, IABP, hemodynamic, endothelial function, endothelial dysfunction

## Abstract

Background: The use of simultaneous veno-arterial extracorporeal membrane oxygenation (ECMO) with or without an Intra-Aortic Balloon Pump (IABP) is a widely used tool for mechanical hemodynamic support. Endothelial function, especially in relation to different cannulation techniques, is rarely investigated in the setting of extracorporeal life support (ECLS). In this study, we analyzed endothelial function in relation to hemodynamic and laboratory parameters for central and peripheral ECMO, with or without concomitant IABP support in a large animal model to gain a better understanding of the underlying basic mechanisms. Methods: In this large animal model, healthy female pigs with preserved ejection fraction were divided into the following groups related to cannulation strategy for ECMO and simultaneous IBAP support: control (no ECMO, no IABP), peripheral ECMO (pECMO), central ECMO (cECMO), pECMO and IABP or cECMO and IABP. During the experimental setting, the blood flow in the ascending aorta, left coronary artery and arteria carotis was measured. Afterwards, endothelial function was investigated after harvesting the right coronary artery, arteria carotis and renal artery. In addition, laboratory markers, such as creatine kinase (CK), creatine kinase muscle–brain (CK-MB), troponin, creatinine and endothelin were analyzed. Results: The blood flow in the ascending aorta and the left coronary artery was significantly lower in all discussed experimental settings compared to the control group. Of note, the cECMO cannulation strategy generated favorable hemodynamic circumstances with higher blood flow in the coronary arteries than pECMO regardless of flow circumstances in the ascending aorta. The concomitant usage of IABP did not result in an improvement of the coronary blood flow, but partially showed a negative impact on the endothelial function of coronary arteries in comparison to the control. These findings correlate to higher CK/CK-MB levels in the setting of cECMO + IABP and pECMO + IABP. Conclusions: The usage of mechanical circulatory support with concomitant ECMO and IABP in a large animal model might have an influence on the endothelial function of coronary arteries while not improving the coronary artery perfusion in healthy hearts with preserved ejection.

## 1. Introduction

The implantation of veno-arterial (VA) extracorporeal membrane oxygenation (ECMO) has become a widely used tool for patients with refractory cardiopulmonary or isolated cardio-circulatory failure and cardiac arrest [1,2,3]. The appropriate cannulation strategy for VA-ECMO application with respect to survival rates and myocardial recovery remains a topic of discussion [4,5,6]. For central access (cECMO) with direct cannulation of the ascending aorta and the right atrium, a sternotomy is mandatory. Peripheral cannulation (pECMO) of the femoral artery and vein is the other predominant implantation technique of VA-ECMO. Further, the simultaneous mechanical support of VA-ECMO with an intra-aortic balloon pump (IABP) leads to significant reduction of intracavitary cardiac pressure, with a better left-ventricular unloading, and generates pulsatile flow conditions [7,8]. Besides the common clinical usage of different VA-ECMO settings, with or without IABP, it is unknown how hemodynamic alterations have an impact on myocardial regeneration and coronary artery endothelial function. Nitric oxide (NO) is a molecule derived from the endothelium of blood vessels orchestrating vascular relaxation in muscular arteries. An endothelial dysfunction, potentially due to mechanical circulatory support via shear stress or circulating inflammatory molecules, can lead to an imbalance and pathologic condition regarding vasodilatation and thrombogenic circumstances. The assessment of NO-mediated vasodilatation provides information about the function and integrity of the endothelium including vascular adaptation [9]. Evaluating the different VA-ECMO therapy settings and related perfusion-mediated endothelial function seems of particular importance to provide a better understanding for cardiac recovery. Consequently, this experimental study was aimed to analyze the effects of different mechanical cardio-circulatory support settings in a healthy porcine model to gain a better understanding for underlying basic mechanisms regarding hemodynamic and endothelial function.

## 2. Materials and Methods

The responsible ethical committee for animal care of the University of Cologne (LANUV Northrhine-Westphalia, Recklinghausen, Germany) approved the study protocol, and the animals were treated in compliance with the Directive 2010/63/EU of the European Parliament. Funding was supported by the Koeln Fortune Programm (343/2015), Faculty of Medicine, University of Cologne. All relevant procedures (experimental protocol and subsequent analysis) were performed between May 2017 and December 2021 at the institution of the Experimental Medicine of the Faculty of Medicine and University Hospital Cologne and the laboratories of the Department of Cardiothoracic Surgery of the University Hospital Cologne [10].

### 2.1. Experimental Setting

Thirty-three healthy female pigs (Deutsche Landrasse Pietrain) with a body weight of 60 kg were divided into four groups: control, peripheral ECMO (pECMO), central ECMO (cECMO), pECMO and IABP or cECMO and IABP. The control group did not receive any cardiac assistance as displayed in Table 1.

Animals were premedicated with combinatory intramuscular application of xylazine (2 mg/kg) and zoletil (10 mg/kg). Additionally, pigs received atropin (0.02 mg/kg i.m.). After initiation of sedation with propofol (2 mg/kg i.v.), animals were intubated endotracheal. Analgo-sedation was maintained with fentanyl (0.012–0.025 mg/kg), midazolam (0.96–1.2 mg/kg) and propofol (4–6 mg/kg) as continuous infusion. Ventilation of pigs was performed using a volume-controlled ventilator (Fabius GS, Dräger, Lübeck, Germany) with the aim to keep arterial blood gas parameters in a physiologic range (pO2 > 100 mmHg). Achievement of full anesthesia was continuously examined using the interdigital claw reflex. After sterile washing and covering, a urinary catheter, a central venous catheter (8.5 Fr, ZVK Arrow International, Reading, PA, USA) through the right jugular vein and an arterial catheter (20 G Leadercath, Vygon, Aachen, Germany) through the right carotid artery were placed surgically. Central venous pressure was kept constant via continuous saline infusion (5–10 mL/kg/h). Mean arterial pressure was adjusted to achieve physiological organ perfusion (>60 mmHg). Moreover, the contralateral carotid artery was used for measurement of cerebral blood flow with transit time flow probes (Transonic Systems Inc., New York, NY, USA). Afterwards, a sternotomy was then performed and the heart was exposed. Further, a pulmonary catheter (20 G Leadercath, Vygon, Aachen, Deutschland) and 5 Fr pressure transducer-tip catheters (Model SPC-350S, Millar Instruments Inc., Houston, TX, USA) were placed into the pulmonary artery and the left and right ventricles. Transit time flow probes (Transonic Systems Inc., New York, NY, USA) recorded cardiac output and coronary blood. Further, the blood flow was recorded by transit time flow probes (Transonic Systems Inc., New York, NY, USA), which were placed at the ascending aorta and the left anterior descending artery, respectively. The right femoral artery and vein were exposed after systemic anticoagulation (heparin 300 IU/kg), and in the case of peripheral ECMO (pECMO) treatment were cannulated in a wire-guided manner. For central cannulation (cECMO), the arterial cannula was directly placed into the ascending aorta. A venous cannula was placed in the right atrium in both scenarios. ECMO circulation was performed for 10 h with a flow index of 50 mL/kg/min/m^2^. ECMO equipment consisted of a console (Bio-Medicus 540, Medtronic, Dublin, Ireland), a centrifugal pump (BPX-80 Bio-Pump, Medtronic, Dublin, Ireland), a membrane oxygenator (CAPIOX^®^ FX, Terumo, Tokyo, Japan), a heat exchanger (Bio-Cal 370, Medtronic, Dublin, Ireland), an extracorporeal circuit (Tubing set, Terumo, Shibuya, Japan) and arterial/venous cannulas (Biomedicus 17 Fr art./21 Fr ven., Medtronic, Dublin, Ireland). IABP (97e, Datascope Corp., Fairfield, NJ, USA) were implanted in the aortic arch and placed distally. After the initiation of extracorporeal circulation, animals were examined for 10 h. Euthanasia was commenced with the application of pentobarbital (80 mg/kg). Measurements were performed and recorded using a 16-channel hemodynamic set-up, all data were digitized at a rate of 500 Hz and were subsequently analyzed (Hugo Sachs Elektronik-Harvard Apparatus GmbH, March-Hugstetten, Germany). Quantification of Arterial blood samples were withdrawn 10 h after ECMO initiation for measurement of parameters of systemic inflammation and endothelial injury (endothelin-1, ET-1). Plasma was separated after centrifugation for 10 min at 2000× *g* rpm and samples were stored at –80 until assay preparation. Enzyme-linked immunosorbent assays were performed in accordance with manufacturers’ recommendations (ET-1, R&D Systems Inc., Minneapolis, MN, USA; MPO, USCN Life Science Inc., Wuhan, China). Further analysis of blood samples, including creatine kinase (CK), creatine kinase muscle–brain (CK-MB), troponin and creatinine, was conducted in the central laboratory of the university hospital (University Hospital Cologne, Cologne, Germany) as previously described by our research group in “*Concomitant Intra-Aortic Balloon Pumping Significantly Reduces Left Ventricular Pressure during Central Veno-Arterial Extracorporeal Membrane Oxygenation—Results from a Large Animal Model*” [10].

### 2.2. In Vitro Endothelial Function

For measurements of endothelial function, we followed an established protocol [11]. Hearts were excised postmortem and the right coronary artery was isolated. Simultaneously, the arteria carotis and arteria renalis were harvested. The arteries were carefully isolated from surrounding tissue and placed in cold (4 °C) modified Krebs–Henseleit solution (143 mmol/L Na^+^, 5.9 mmol/L K^+^, 1.6 mmol/L Ca^2+^, 1.2 mmol/L Mg^2+^, 126 mmol/L Cl^−^, 25 mmol/L HCO_3_^−^, 1.2 mmol/L H_2_PO_4_^−^, 1.2 mmol/L SO_4_^2−^, 5.1 mmol/L glucose). For measurements, vessels were cut transversely into rings (4 mm width) using a no-touch technique. For each run, 4 rings of the specific artery were mounted on steel hooks with a constant load of 1 g and incubated in isolated organ baths (Hugo Sachs Elektronik) with 10 mL preoxygenated Krebs–Henseleit buffer at 37 °C and 95% O_2_/5% CO_2_. After equilibration for 30 min, isotonic alterations of vascular ring diameter were constantly recorded (Multipen Recorder; Rikadenki Kogyo Co., Tokyo, Japan). Potassium chloride (60 mmol/L) was added to trigger a maximal contraction and rinsed three times. Indomethacin (10 μmol/L) was used to block the cyclooxygenase pathway (prostaglandin synthesis). Either L-ARGININ or N-nitro-L-arginine (LNNA, 300 μmol/L, Sigma-Aldrich, Burlington, MA, USA) was added to induce or block NO production, respectively. Stable precontraction was then induced by prostaglandin F2_a_ (10 μmol/L) and endothelium-dependent relaxation to bradykinin was recorded by adding accumulating concentrations (0.0001–10 μmol/L). The mean vessels endothelial function was determined by calculating the average percentage change of vasorelaxation following bradykinin administration [10].

### 2.3. Statistical Analysis

Statistical analyses were performed with GraphPad-Prism9 software (GraphPad Software, La Jolla, CA, USA). The data are presented as (1) boxes with violins (min to max) for flow measurements; (2) points and connection line with error bars (mean and error ± standard error of the mean) for measurement of the endothelial function as loss of contraction; and (3) scatter plots with bars (mean ± standard error of the mean) for laboratory analysis. Data were analyzed using one-way ANOVA or two-way ANOVA with Tukey’s multiple comparisons test for comparison of more than two groups. Intergroup differences between two groups were analyzed using the unpaired Student’s *t*-test. A *p*-value of less than 0.05 was considered statistically significant. 

## 3. Results

Four main settings with respect to different canulation strategies (peripheral or central) and ± IABP assistance were compared: (1) pECMO versus cECMO; (2) pECMO + IABP versus cECMO + IABP; (3) pECMO versus pECMO + IABP; and (4) cECMO versus cECMO + IABP. Within every setting, results were compared to the control group (no ECLS). Groups were compared regarding the blood flow in the ascending aorta (A) and coronary artery (B), endothelial function in the coronary artery (C + D), heart enzymes (E–G) and endothelin (H).

### 3.1. Comparison of Peripheral and Central ECMO

**Figure 1:**
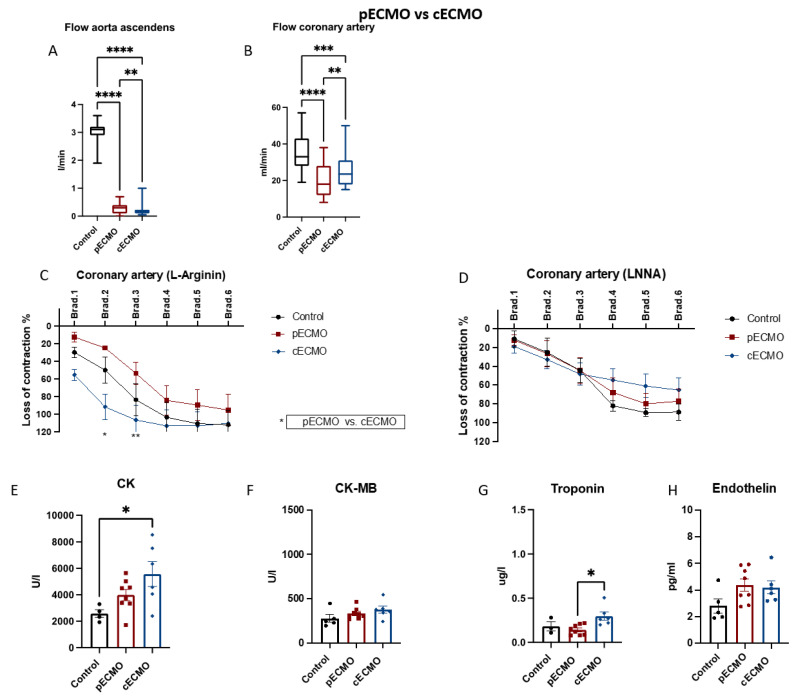
Peripheral and central ECMO cannulation showed differences in aorta ascendens and coronary artery flow profiles with impact on NO-dependent vasodilation. Female swine were treated with either peripheral ECMO (pECMO), central ECMO (cECMO) or as control group (control) for 10 h. Measuring of flow in the aorta ascendens after implantation of ECMO (30 min and every hour from 1–10) and left anterior descending coronary artery (every hour from 5–10) in vivo. Examination of coronary artery endothelial function ex vivo after euthanasia of swine. (**A**) Flow in the aorta ascendens L/min (n = 5, 8, 6) and (**B**) flow measurement in the coronary artery mL/min (n = 5, 8, 6) (**C**) NO-dependent (L-Arginin) measurement of endothelial function (n = 3, 3, 3) (**D**) NO-independent measurement of endothelial function (n = 4, 8, 7). Measuring of creatinine kinase (CK), creatinine kinase muscle–brain (CK-MB) and troponin at the end of ECMO therapy (10 h), (**E**) CK U/L (n = 5, 8, 6) and (**F**) CK-MB U/L (n = 5, 8, 6), (**G**) Troponin ug/L (n = 5, 8, 6) and (**H**) Endothelin pg/mL (n = 5, 8, 6). Data are presented as boxes with violins (min to max) for (**A**) + (**B**) or as points and connection lines with error bars (mean and error ± SEM) for (**C**) + (**D**); (**E**–**H**) scatter plots with bars (mean ± SEM). One-way ANOVA ((**A**) + (**B**), (**E**–**H**)) or two-way ANOVA (**C**) + (**D**) with Tukey’s multiple comparisons test: * *p* < 0.05, ** *p* < 0.01, *** *p* < 0.001, **** *p* < 0.0001.

Peripheral and central ECMO cannulation revealed differences regarding blood flow in the aorta ascendens with 0.3 L/min (0.0–0.7 L/min) in the pECMO and 0.2 L/min (0.1–1.0 L/min) in the cECMO group (*p* < 0.01) as displayed in Figure 1a. The control group showed a physiological flow of 3.0 L/min (1.9–3.6 L/min), yielding significant differences in comparison to the pECMO and cECMO group (*p* < 0.0001). Blood flow in the coronary artery was significantly reduced in the ECMO groups when compared to control (35, 19–57 mL/min), *p* < 0.001 and *p* < 0.0001. cECMO showed a significantly higher coronary perfusion of (26, 15–50 mL/min) compared to the pECMO group (19, 8–38 mL/min) with *p* < 0.01 (Figure 1b). Further comparisons for intergroup flow measurements of aorta ascendens and coronary artery are displayed in supplemental material (Supplementary Figure S1a,b). Right coronary NO-dependent endothelial-derived relaxation (EDR) was significantly improved (Figure 1c) in the cECMO (98 ± 9%) setting when compared to pECMO (60 ± 14%), showing a positive correlation to coronary artery perfusion of these groups (control 82 ± 14%). In contrast, NO-independent EDR (Figure 1d) was not affected by different ECMO settings since vasorelaxation by bradykinin was similar in all groups (control 57 ± 14%, pECMO 51 ± 11%, cECMO 47 ± 7%). Creatinine kinase (CK) 5570 ± 940 versus 3985 ± 431 U/L and creatinine kinase muscle–brain (CK-MB) 374 ± 40 versus 333 ± 23 U/L were comparable between cECMO and pECMO after 10 h. CK was significantly (*p* < 0.05) higher in the cECMO group when compared to control (5570 ± 940 versus 3426 ± 878 U/L). Troponin was elevated with 0.3 ± 0.05 μg/L in the cECMO group compared to pECMO (0.14 ± 0.02 μg/mL, *p* < 0.05), control 0.18 ± 0.05 μg/L. Endothelin did not differ between groups after 10 h of ECMO therapy (control 2.8 ± 0.5 pg/mL, pECMO 4.4 ± 0.5 pg/mL and cECMO 4.2 ± 0.5 pg/mL), as shown in Figure 1e–h. Of note, endothelin baseline levels and measurements compared to control and after 10 h of pECMO and cECMO were comparable, but intragroup comparison of endothelin levels in the cECMO group showed elevated levels at the end of therapy (Supplementary Figure S2a–c,f,g). Analyses of the blood flow and endothelial function of the arteria carotis (Supplementary Figure S3a–c) show a higher perfusion under cECMO therapy (202, 101–292 mL/min) when compared to control (160, 102–290 mL/min), *p* < 0.001. Comparing pECMO (171, 105–297 mL/min) and cECMO also revealed significantly higher blood flow under cECMO therapy, *p* < 0.001. NO-dependent EDR was significantly improved in both ECMO settings compared to the control group (control: 6 ± 8%, pECMO 41 ± 20%, cECMO 43 ± 16%, *p* < 0.05). There were no differences in NO-independent EDR measurements. Examination of the renal artery EDR revealed no differences between groups and creatinine serum levels were comparable (Supplementary Figure S4a–c).

### 3.2. Comparison of Peripheral and Central ECMO with IABP

**Figure 2:**
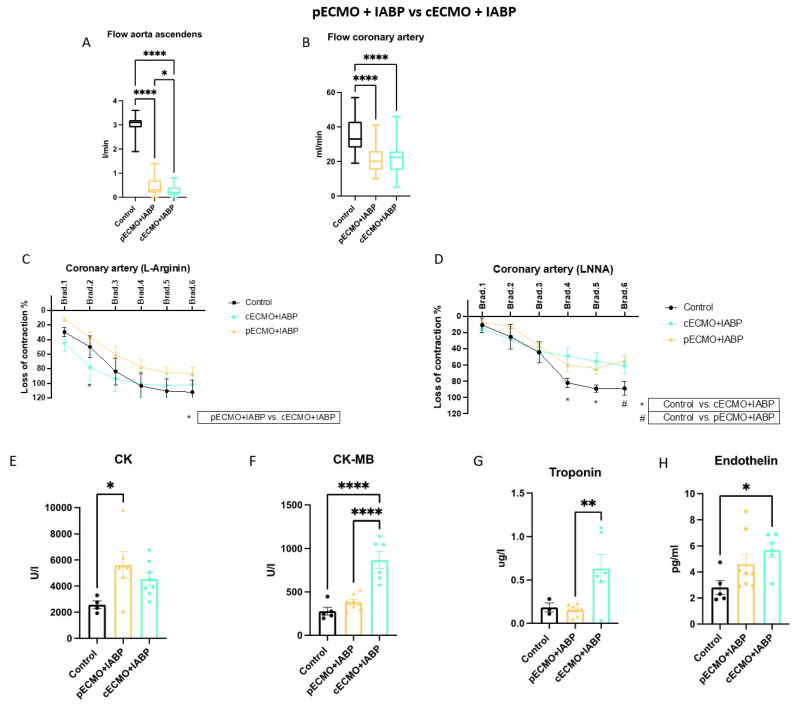
Peripheral and central ECMO with IABP show differences in aorta ascendens and coronary artery flow profiles with impact on NO-dependent vasodilation. Female swine were treated with either peripheral ECMO (pECMO) and intra-aortic balloon pump (IABP), central ECMO (cECMO) and IABP or as control group (control) for 10 h. Measuring of flow in the aorta ascendens after implantation of ECMO and IABP (30 min and every hour from 1–10) and left anterior descending coronary artery (every hour from 5–10) in vivo. Examination of coronary artery endothelial function ex vivo after euthanasia of swine. (**A**) Flow in the aorta ascendens L/min (n = 5, 8, 6) and (**B**) flow measurement in the coronary artery mL/min (n = 5, 8, 6), (**C**) NO-dependent (L-Arginin) measurement of endothelial function (n = 3, 4, 6) and (**D**) NO-independent measurement of endothelial function (n = 4, 9, 12). Measuring of creatinine kinase (CK), creatinine kinase muscle–brain (CK-MB) and troponin at the end of ECMO therapy (10 h) (**E**) CK U/L (n = 5, 8, 6) and (**F**) CK-MB U/L (n = 5, 8, 6), (**G**) Troponin ug/L (n = 5, 8, 6) and (**H**) Endothelin pg/mL (n = 5, 8, 6). Data are presented as boxes with violins (min to max) for (**A**) + (**B**) or as points and connection lines with error bars (mean and error ± SEM) for (**C**) + (**D**); and (**E**–**H**) scatter plots with bars (mean ± SEM). One-way ANOVA ((**A**) + (**B**), (**E**–**H**)) or two-way ANOVA (**C**) + (**D**) with Tukey’s multiple comparisons test: * *p* < 0.05, ** *p* < 0.01, **** *p* < 0.0001.

Blood flow in the aorta ascendens differed significantly between the control (3.0, 1.9–3.6 L/min) and pECMO + IABP (0.4, 0.0–1.4 L/min) or cECMO + IABP (0.3, 0.0–0.8 L/min) *p* < 0.001. Similarly, coronary artery perfusion in the pECMO + IABP (21, 10–41 mL/min) and cECMO + IABP (21, 5–46 mL/min) was lower when compared to control (35, 19–27 mL/min) *p* < 0.001. A significant flow difference in the aorta ascendens between both ECMO + IABP groups showed no correlation to the coronary artery perfusion (Figure 2a,b). NO-dependent EDR of the right coronary artery was significantly (*p* < 0.05) improved in the cECMO + IABP (87 ± 23%) versus pECMO + IABP (60 ± 30%) groups, but not when compared to control (82 ± 35%) (Figure 2c). NO-independent EDR (Figure 2d) did not improve in the cECMO + IABP (42 ± 17%) and pECMO + IABP (40 ± 25%) groups and showed significantly better reactions in the control group (57 ± 35%), *p* < 0.05. Laboratory parameters (Figure 2e–g) showed higher CK in the pECMO + IABP setting (5613 ± 1021 U/L) when compared to control (3426 ± 878 U/L) with *p* < 0.05, but not for cECMO + IABP (4538 ± 463 U/L). CK-MB was significantly elevated in the pECMO + IABP (382 ± 31 U/L) and cECMO + IABP (868 ± 98 U/L) groups compared to the control group (278 ± 44 U/L), *p* < 0.0001. Troponin differed between the pECMO + IABP (0.2 ± 0.02 μg/L) and cECMO + IABP (0.6 ± 0.2 μg/L) groups, *p* < 0.01 (Control: 0.2 ± 0.05 μg/L). Further, endothelin was highest in the cECMO + IABP setting (5.7 ± 0.6 pg/mL) and differed significantly in comparison to control (2.8 ± 0.5 pg/mL), *p* < 0.05 (pECMO + IABP: 4.6 ± 0.8 pg/mL).

Blood flow in the arteria carotis (Supplementary Figure S5a) was comparable between the control (162, 102–290 mL/min) and pECMO + IABP (161, 110–346 mL/min) groups, but was significantly higher in the cECMO + IABP (228, 135–411 mL/min) group, *p* < 0.001. NO-dependent EDR (Supplementary Figure S5b) improved significantly (*p* < 0.05) in the pECMO + IABP (40 ± 21%) group in comparison to control (6 ± 8%), but not in the cECMO + IABP group (42 ± 16%). In comparison, NO-independent EDR (Supplementary Figure S5c) was better in the control (47 ± 19%) and cECMO + IABP (43 ± 14%) groups when compared to pECMO + IABP (17 ± 11%). NO-dependent EDR for the arteria renalis was comparable between groups (Supplementary Figure S6a), but differed significantly for the control (42 ± 36%) and pECMO + IABP (35 ± 23%) groups in the NO-independent (Supplementary Figure S6b) measurements, *p* < 0.05 (cECMO + IABP: 45 ± 23%). Serum creatinine was significantly higher in the cECMO + IABP (2.9 ± 0.3 mg/dL) when compared to control (1.4 ± 0.1 mg/dL) and pECMO + IABP (1.7 ± 0.1 mg/dL), *p* < 0.001.

### 3.3. Peripheral ECMO with and without IABP

**Figure 3:**
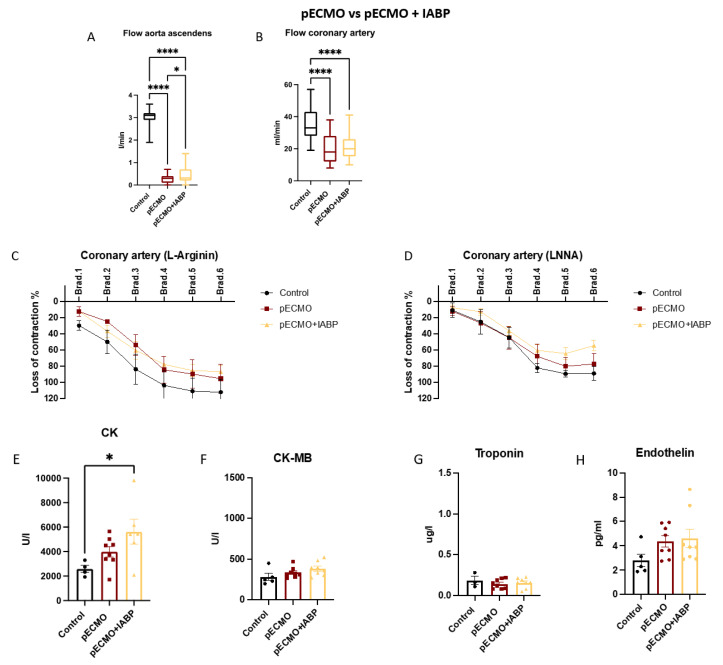
Peripheral ECMO with IABP shows no differences in coronary artery flow profiles when compared to peripheral ECMO without IABP. Endothelial function between groups were comparable. Female swine were treated with either peripheral ECMO (pECMO) and intra-aortic balloon pump (IABP), pECMO or as control group (control) for 10 h. Measuring of flow in the aorta ascendens after implantation of ECMO and IABP (30 min and every hour from 1–10) and left anterior descending coronary artery (every hour from 5–10) in vivo. Examination of coronary artery endothelial function ex vivo after euthanasia of swine. (**A**) Flow in the aorta ascendens L/min (n = 5, 8, 8) and (**B**) flow measurement in the coronary artery mL/min (n = 5, 8, 8), (**C**) NO-dependent (L-Arginin) measurement of endothelial function (n = 3, 3, 6) and (**D**) NO-independent measurement of endothelial function (n = 4, 8, 12). Measuring of creatinine kinase (CK), creatinine kinase muscle–brain (CK-MB) and troponin at the end of ECMO therapy (10 h), (**E**) CK U/L (n = 5, 8, 8) and (**F**) CK-MB U/L (n = 5, 8, 8), (**G**) Troponin ug/L (n = 5, 8, 8) and (**H**) Endothelin pg/mL (n = 5, 8, 8). Data are presented as boxes with violins (min to max) for (**A**) + (**B**) or as points and connection lines with error bars (mean and error ± SEM) for (**C**) + (**D**); and (**E**–**H**) scatter plots with bars (mean ± SEM). One-way ANOVA ((**A**) + (**B**), (**E**–**H**)) or two-way ANOVA (**C**) + (**D**) with Tukey’s multiple comparisons test: * *p* < 0.05, **** *p* < 0.0001.

The blood flow in the aorta ascendens (Figure 3a) was significantly lower for pECMO (0.3, 0.0–0.7 L/min) and pECMO + IABP (0.4, 0.0–1.4 L/min) in comparison to the control group (3.0, 1.9–3.6 L/min), *p* < 0.001. An intergroup comparison showed a higher flow for pECMO + IABP when compared to pECMO alone (*p* < 0.05). In correlation to the measured aorta ascendens flow, coronary artery perfusion (3b) differed between control (35, 19–57 mL/min) and pECMO (19, 8–38 mL/min) or pECMO + IABP (21, 10–41 mL/min), both *p* < 0.0001. EDR, No-dependent and NO-independent, revealed no differences between settings (Figure 3c,d). Blood analysis revealed a higher CK for pECMO + IABP (5613 ± 1021 U/L) versus control (3426 ± 878 U/L), *p* < 0.05. There were no CK differences between pECMO (3985 ± 431 U/L) and pECMO + IABP. Further, CK-MB, troponin and endothelin were comparable between control, pECMO and pECMO + IABP (Figure 3e–h).

### 3.4. Central ECMO with or without IABP

**Figure 4:**
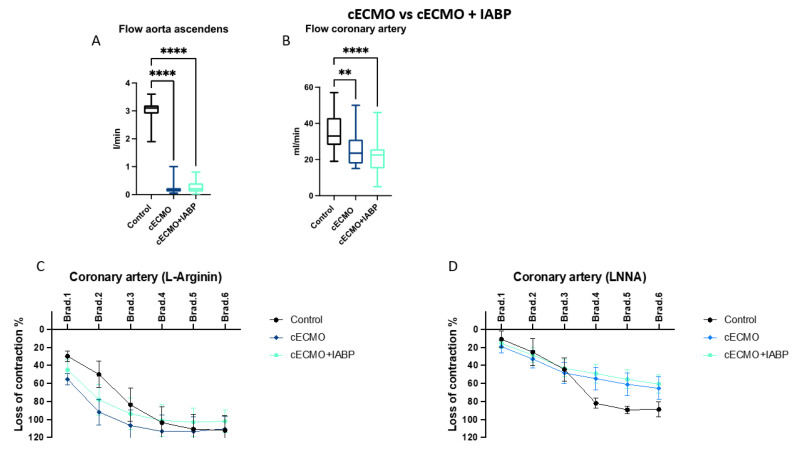

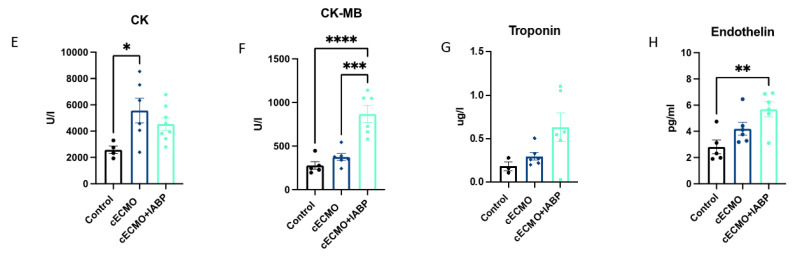
Central ECMO with IABP shows no differences in coronary artery flow profiles when compared to central ECMO without IABP. Endothelial function between groups were comparable. Female swine were treated with either central ECMO (cECMO) and intra-aortic balloon pump (IABP), cECMO or as control group (control) for 10 h. Measuring of flow in the aorta ascendens after implantation of ECMO and IABP (30 min and every hour from 1–10) and left anterior descending coronary artery (every hour from 5–10) in vivo. Examination of coronary artery endothelial function ex vivo after euthanasia of swine. (**A**) Flow in the aorta ascendens L/min (n = 5, 6, 6) and (**B**) flow measurement in the coronary artery mL/min (n = 5, 6, 6), (**C**) NO-dependent (L-Arginin) measurement of endothelial function (n = 3, 3, 4) and (**D**) NO-independent measurement of endothelial function (n = 4, 7, 9). Measuring of creatinine kinase (CK), creatinine kinase muscle–brain (CK-MB) and troponin at the end of ECMO therapy (10 h), (**E**) CK U/L (n = 5, 6, 6) and (**F**) CK-MB U/L (n = 5, 6, 6), (**G**) Troponin ug/L (n = 5, 6, 6) and (**H**) Endothelin pg/mL (n = 5, 6, 6). Data are presented as boxes with violins (min to max) for (**A**) + (**B**) or as points and connection lines with error bars (mean and error ± SEM) for (**C**) + (**D**); and (**E**–**H**) scatter plots with bars (mean ± SEM). One-way ANOVA ((**A**) + (**B**), (**E**–**H**)) or two-way ANOVA (**C**) + (**D**) with Tukey’s multiple comparisons test: * *p* < 0.05, ** *p* < 0.01, *** *p* < 0.001, **** *p* < 0.0001.

The measured flow in the aorta ascendens differed between the control (3.0, 1.9–3.6 L/min) and cECMO (0.2, 0.1–1.0 L/min) or cECMO + IABP (0.3, 0.0–0.8 L/min) groups, both *p* < 0.0001. Similarly, coronary artery blood flow was significantly lower in the cECMO (26, 15–50 mL/min) group when compared to control (35, 19–57 mL/min) with *p* < 0.01 and in cECMO + IABP (21, 5–46 mL/min) with *p* < 0.0001, respectively (Figure 4a,b). No differences in the EDR were seen between groups (Figure 4c,d). CK measurements showed higher values in the cECMO group (5570 ± 940 U/L) versus control (3426 ± 878 U/L), *p* < 0.05 (cECMO + IABP: 4538 ± 468 U/L). Interestingly, CK-MB did not differ between the control (278 ± 44 U/L) and cECMO (374 ± 40 U/L) groups, but for both when compared to cECMO + IABP (868 ± 98 U/L), *p* < 0.001 and *p* < 0.0001 (Figure 4e,f). Troponin levels were comparable, with no intergroup differences (Figure 4g). Endothelin was higher in the cECMO + IABP setting (5.7 ± 0.6 pg/mL) compared to control (2.8 ± 0.5 pg/mL), *p* < 0.01 (cECMO: 4.2 ± 0.5 pg/mL) (Figure 4h).

## 4. Discussion

Our study significantly contributes to the understanding and delineation of the physiological and pathophysiological processes taking place under mechanical circulatory support.

The main findings of this study are: (I) An improved NO-dependent EDR of the coronary artery through a better perfusion under cECMO therapy. (II) Simultaneous IABP support in addition to ECMO leads to a comparable coronary artery perfusion without showing any benefit regarding the NO-dependent EDR. (III) Perfusion of the arteria carotis is significantly improved in cECMO compared to pECMO therapy, independent of simultaneous IABP support. (IV) Different mechanical circulatory support settings showed no impact on renal artery EDR in our pig model. 

### 4.1. Endothelial Function

Endothelial injury and dysfunction following cardio-pulmonary bypass can cause vascular cell impairment and end-organ damage triggered by acute inflammatory response [12]. Exposure of blood to the extracorporeal circuit leads to contact system activation and promotes humoral and cellular inflammation, mimicking systemic inflammatory response syndrome (SIRS) [13]. Endothelial dysfunction, mediated by SIRS, plays a major role in critical illness and is associated with higher mortality [14,15]. Most investigations regarding endothelial function have been assessed in cardio-pulmonary bypass of patients undergoing cardiac surgery, and systematic investigations on this topic for mechanical circulatory support with ECMO are lacking. Therefore, we are the first to systematically investigate the different perfusion profiles of pECMO ± IABP and cECMO ± IABP settings with regard to NO-related endothelial function in the relevant end organs. In this study, we show a positive impact of central versus peripheral cannulation for ECMO implantation on coronary NO-dependent endothelial-derived relaxation. This finding is in line with an improved coronary perfusion in the cECMO setting. Interestingly, clinical experiences with a centrally implanted mechanical life-support system did not outperform peripheral implanted ECMO in patients with or without post-cardiogenic shock (PCS) when assessed for weaning from ECMO or all-cause mortality [1,16]. The addition of an IABP to either cECMO or pECMO circulatory support reduced the coronary artery perfusion and the positive impact of central versus peripheral ECMO cannulation on EDR declined in the discussed experiments. Further, higher serum levels of cardiac markers were detected in the ECMO + IABP groups, representing a potential myocardial damage. Nevertheless, these findings might be due to the effect of an unphysiological blood flow mediated by the ECLS in combination with the experimental setting of healthy hearts and unaffected left-ventricular ejection fraction. In contrast, some clinical studies showed a higher weaning rate from ECMO for cardiogenic shock patients with concomitant IABP circulatory support but contrary outcomes regarding short-term mortality [7,8,17,18]. Differentiation of the patient collective unveils that patients with a recent myocardial infarction under ECMO support might experience higher benefit from additional IABP implementation than those without [19]. 

### 4.2. Hemodynamic Assessment

Besides the endothelial function, our study group assessed the different canulation strategies for ECMO therapy and the concomitant mechanical support via IABP with respect to hemodynamic outcomes. Nevertheless, there is an ongoing debate as to whether ECMO therapy should be supported by an IABP or not, as the concept of ventricular unloading with simultaneous IABP has already been described and is frequently used, although IABP support is an indirect method used to unload ventricular cavities. Our study group also demonstrated an analysis of n = 172 ECMO patients with post-cardiotomy cardiogenic shock showing that, independent of ECMO type or canulation strategy, additional IABP support might increase weaning rates off ECLS support with ECMO without affecting in-hospital mortality rates [8]. Furthermore, Madershahian et al. showed that pulsatility induced by IABP improved diastolic filling index and mean coronary bypass graft flows by reducing coronary vascular resistance during peripheral ECMO in post-cardiotomy cardiogenic shock patients [20]. In contrast, our presented data did not show an increase in coronary or carotid blood flow with the additional usage of an IABP in healthy hearts of pigs. Further, the reduced blood flow in the coronary arteries compared to control (but not for the cECMO group) may be a result of the repetitive aortic occlusion induced by the balloon and the unphysiological retrograde flow in peripheral canulated ECMO settings, diminishing the blood flow available for the aortic root. However, the setting with healthy animals and no reduction in cardiac function may have led to a bias that perfusion of the carotidal arteries was better with cECMO, regardless of simultaneous IABP support, when compared to peripheral cannulation. These findings are in line with the prior literature confirming better hemodynamic and O2 supply to the brain [21]. Nevertheless, there exists no general statement when interpreting the flow modularity of ECLS with ECMO ± IABP as the cardiac output and the lung function plays a crucial interfering role. Hence, only an idealized interpretation with regard to flow patterns and flow distribution can be assessed [22].

## 5. Limitations

The artificial scenario implanting ECMO and IABP in healthy pigs does not represent a real-life scenario. Moreover, our study protocol in healthy pigs did not aim to produce a pathologic state. Therefore, the investigated data are difficult to transfer onto clinical situations. However, our focus was the hemodynamic and endothelial changes caused by different strategies of extracorporeal life support so as to gain a better understanding of the basic mechanisms in these settings of ECLS with ECMO and/or IABP. Further experiments with ischemic hearts or in a setting of ischemia and reperfusion are needed to systematically asses the influence of the discussed ECLS strategies after a cardiac event. In this regard, all results should be viewed with caution, and transfer of these results into a human clinical scenario might be difficult. The examined animal model, with only a small number of pigs authorized for investigation, limits this study. It remains unclear whether findings can be transferred in a 1:1 manner to human patients. Further, when interpreting these data, the distinct experimental setting has to be considered in terms of an unaffected and preserved ejection fraction. Therefore, the comparison of the used animal model may only partially reflect the hemodynamic settings of humans in need of extracorporeal mechanical support with PCS or acute heart failure. In addition, the impaired blood flow in the ascending aorta can only partially explained by an unphysiological flow produced by the ECLS, but was validated by re-positioning of the flow measurement and does not reflect the overall perfusion generated by ECMO as this was computed and determined beforehand. The study was not designed to analyze outcome measures, and the design of the study does not allow a reasonable interpretation of the reported results. In order to analyze the background and underlying mechanisms of our findings, further experiments are needed, including studies with ischemic hearts and/or reduced ejection fraction. As for now, this study can only be considered an observational study.

## 6. Conclusions

Mechanical circulatory support with ECMO and plus/minus IABP might have an impact on the endothelial function, as investigated in this large animal model. Further, hemodynamic measurements showed conflicting results while using mechanical circulatory support. In order to properly assess the impact of ECMO and concomitant IABP support, long-term experimental settings are needed.

## Figures and Tables

**Table 1:** Experimental settings with different ECLS strategies.

Experimental Settings					
Group	Control	pECMO	cECMO	pECMO and IABP	cECMO and IABP
Type of mechanical support	None	ECMO with peripheral canulation	ECMO with central canulation	ECMO with peripheral canulation plus IABP	ECMO with central canulation plus IABP

## Data Availability

Data supporting reported results can be provided on request of the corresponding author.

## Supplementary Materials

The following supporting information can be downloaded at: https://www.mdpi.com/article/10.3390/jcm12124038/s1, Figure S1: Flow measurements; Figure S2: Endothelin; Figure S3: pECMO vs cEcmo; Figure S4: pECMO vs Cecmo; Figure S5: pECMO + IABP vs cECMO + IABP; Figure S6: pECMO + IABP vs cECMO + IABP.
