# Supplementary material for: Endothelial and Hemodynamic Function in a Large Animal Model in Relation to Different Extracorporeal Membrane Oxygenation Cannulation Strategies and Intra-Aortic Balloon Pumping
Source: J Clin Med. 2023 Jun 13;12(12):4038. doi: 10.3390/jcm12124038 (PMC10299098; doi:10.3390/jcm12124038)
Supplement: Supplementary file 1 [file jcm-12-04038-s001.zip › jcm-2393743-supplementary.pdf]

Supplementary figures

Figure S1

Flow measurements – multiple comparison

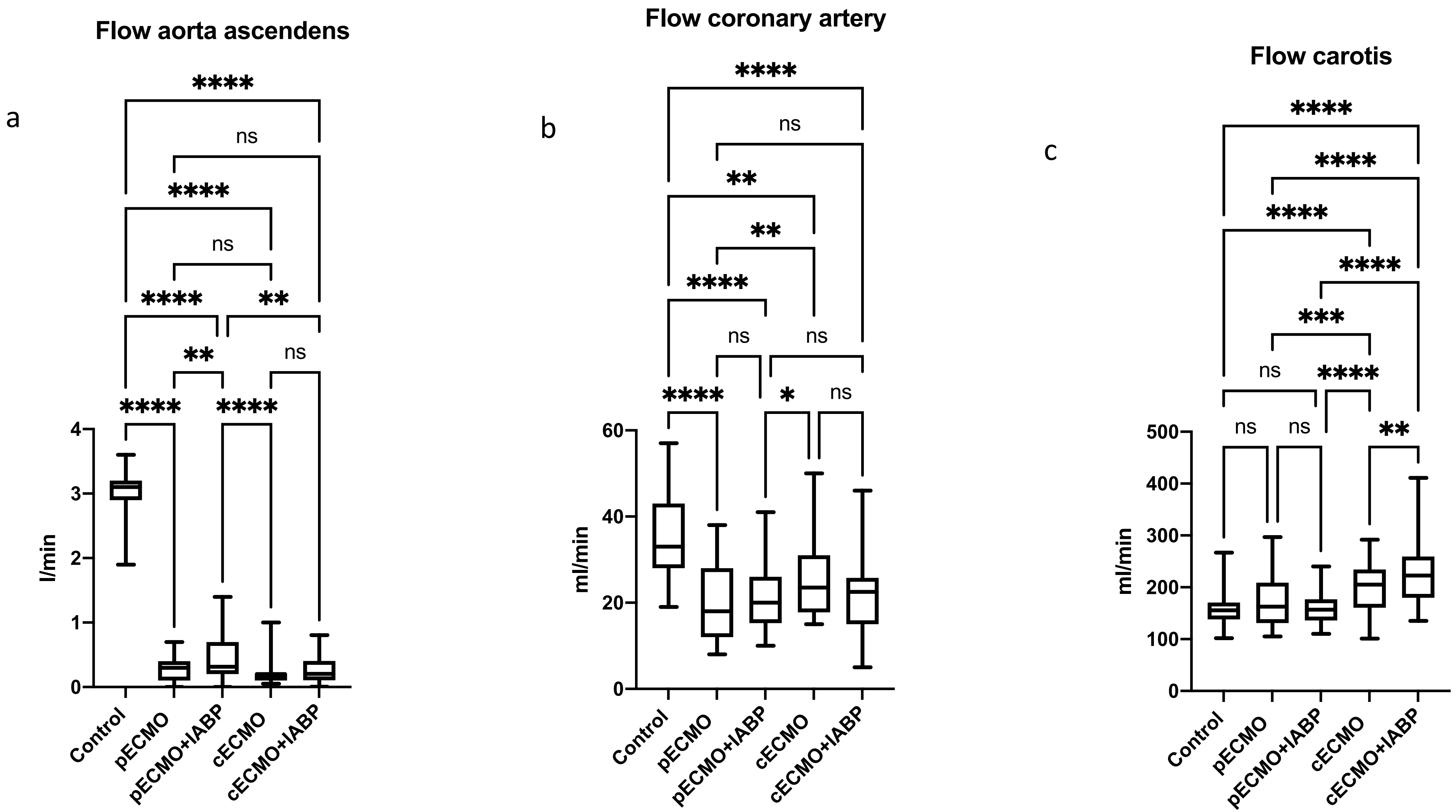

(A) Flow in the aorta ascendens l/min, (B) flow measurement in the coronary artery ml/min and (C) flow measurement in the carotid artery; Data are presented as boxes with violins (min to max) .
(B) One-way ANOVA: * $p < 0.05$, ** $p < 0.01$, *** $p < 0.001$, **** $p < 0.0001$.

Figure S2

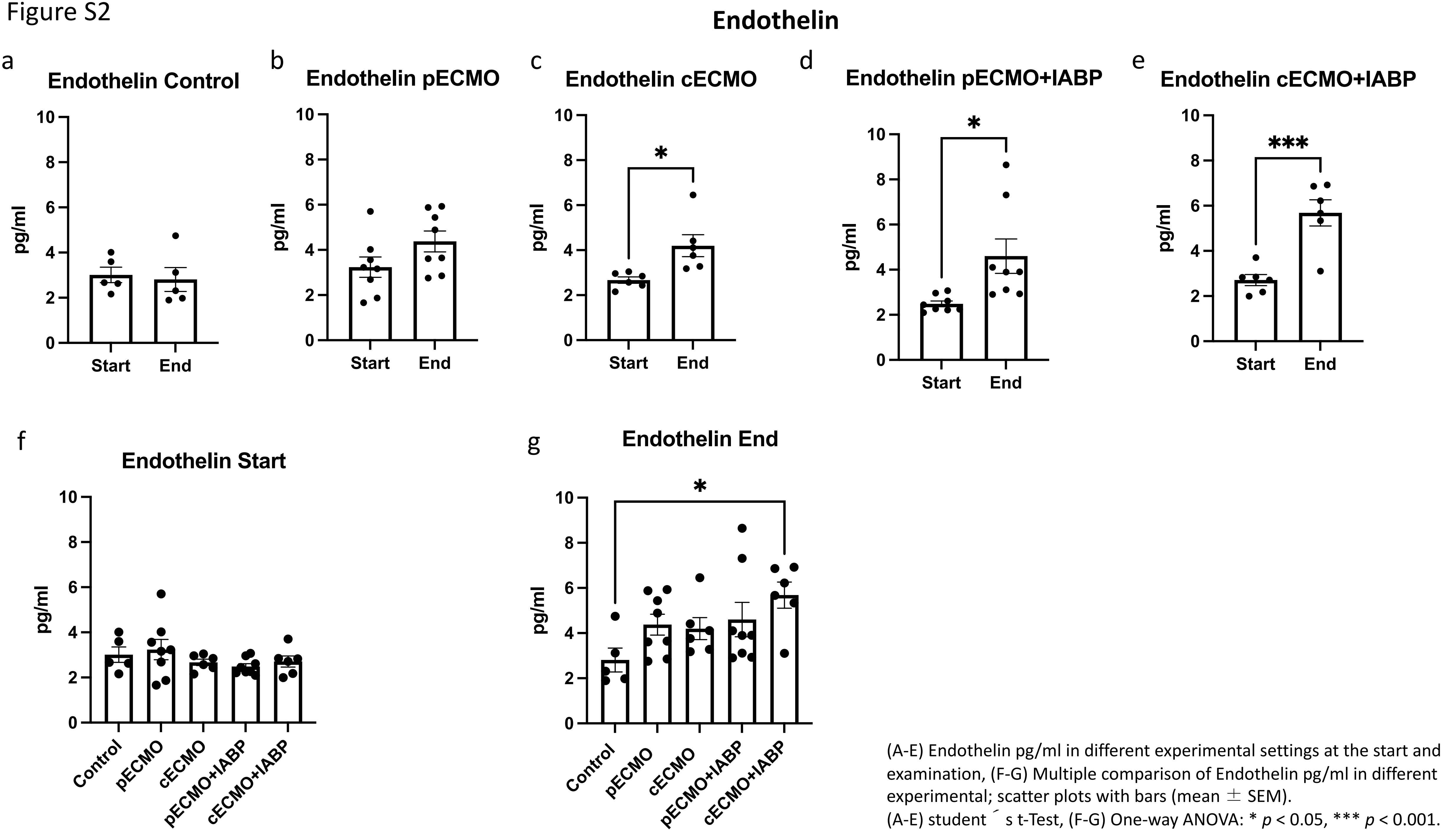

(A-E) Endothelin pg/ml in different experimental settings at the start and end of examination, (F-G) Multiple comparison of Endothelin pg/ml in different experimental; scatter plots with bars (mean $\pm$ SEM).
(A-E) student´s t-Test, (F-G) One-way ANOVA: * $p < 0.05$, *** $p < 0.001$.

Figure S3

pECMO vs cECMO

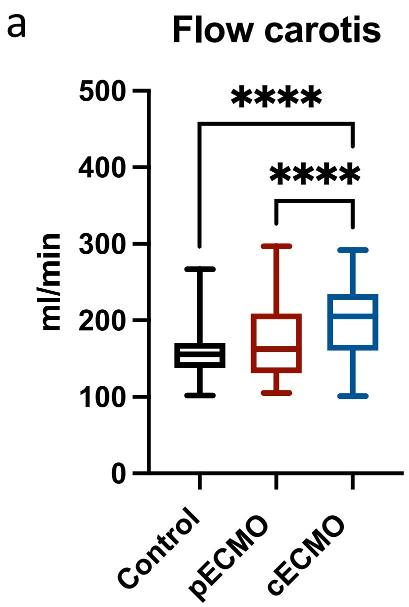

(A) flow measurement in the coronary artery ml/min, (B) NO-dependent (L-Arginin) measurement of (B) endothelial function and (C) NO-independent measurement of endothelial function. Data are presented as boxes with violins (min to max) (A) or as points and connection lines with error bars (mean and error ± SEM) for (B-C); One-way ANOVA (A) or two-way ANOVA (B-C)) with Tukey´s multiple comparisons test: * $p < 0.05$, **** $p < 0.0001$.

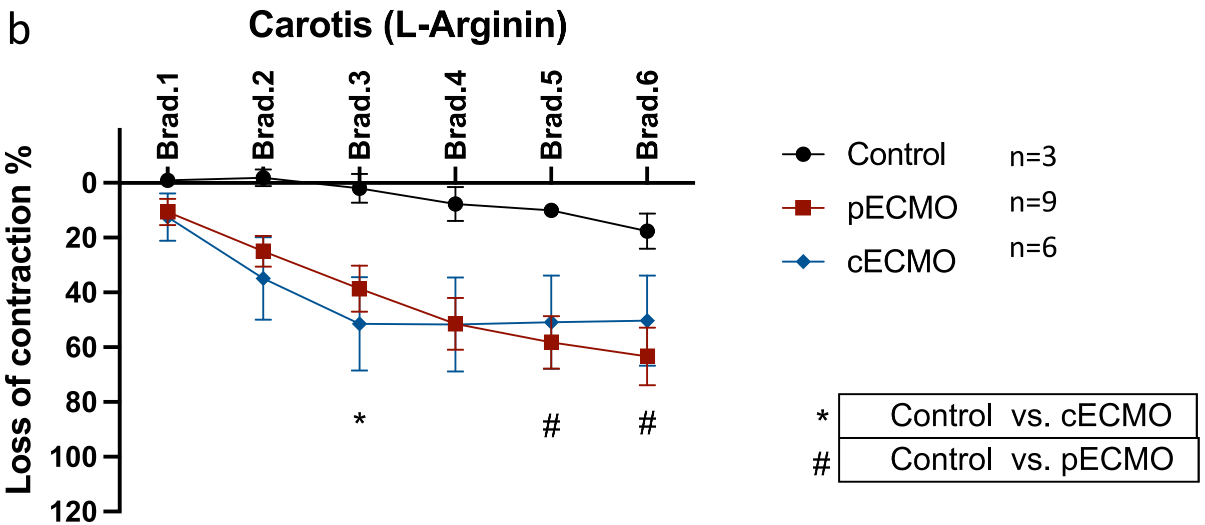

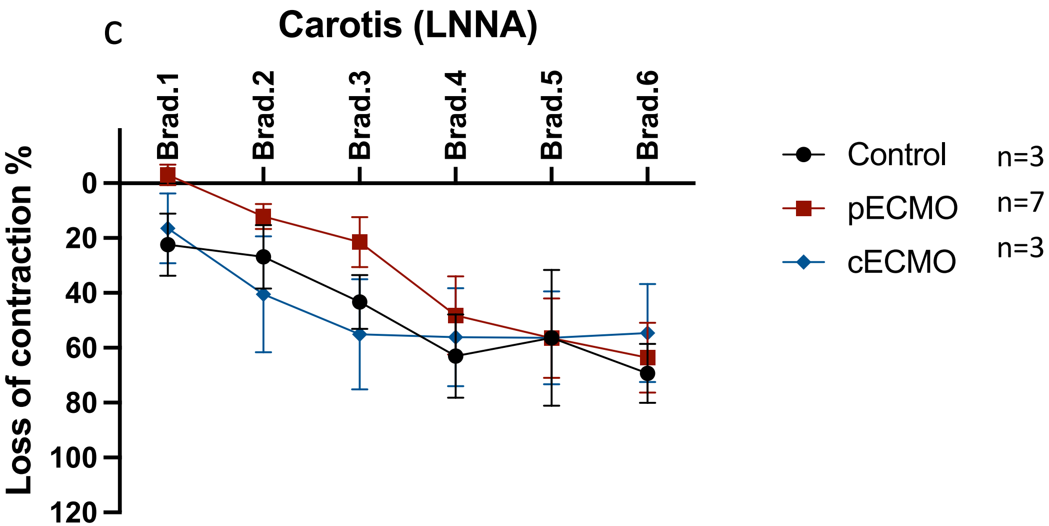

Figure S4

pECMO vs cECMO

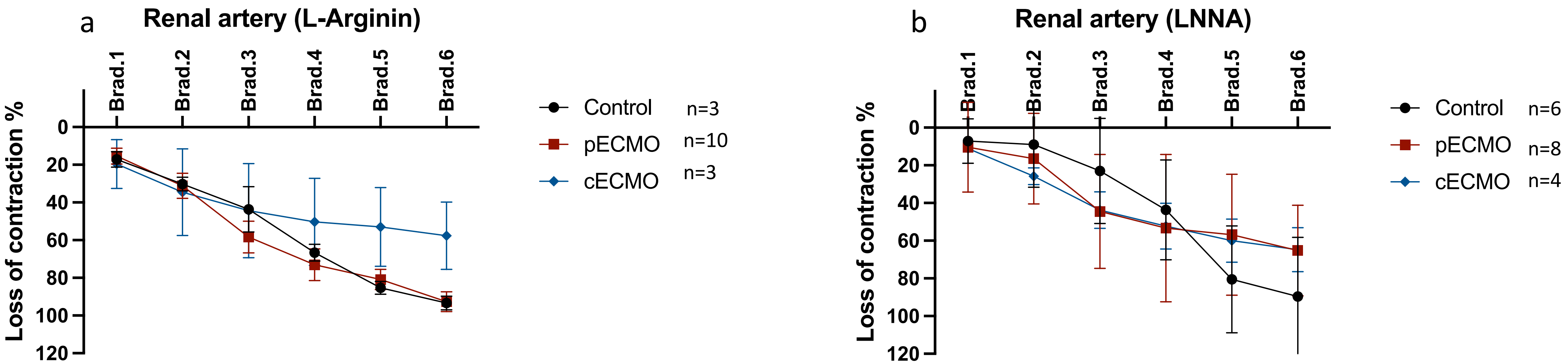

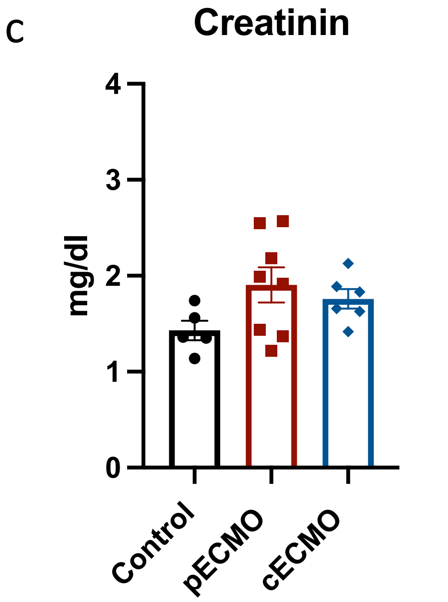

(A) NO-dependent (L-Arginin) measurement of endothelial function and (B) NO-independent measurement of endothelial function. (C) Creatinin in mg/dl; Data are presented as points and connection lines with error bars (mean and error ± SEM) for (A-B) or as scatter plot´s with error bars (C); Two-way ANOVA with Tukey´s multiple comparisons test or One-way ANOVA

Figure S5

pECMO+IABP vs cECMO+IABP

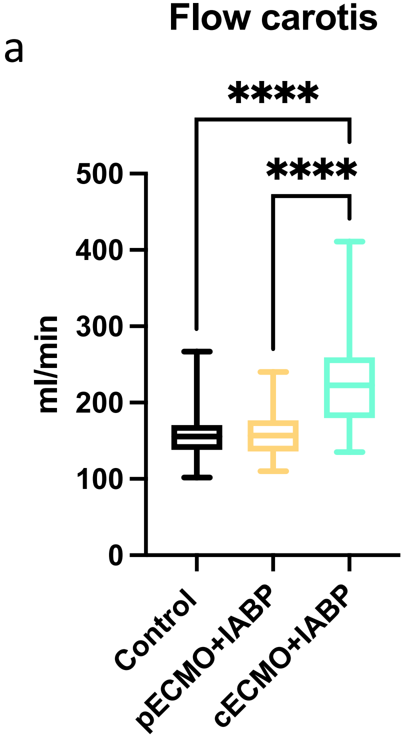

(A) flow measurement in the coronary artery ml/min, (B) NO-dependent (L-Arginin) measurement of (B) endothelial function and (C) NO-independent measurement of endothelial function. Data are presented as boxes with violins (min to max) (A) or as points and connection lines with error bars (mean and error ± SEM) for (B-C); One-way ANOVA (A) or two-way ANOVA (B-C)) with Tukey´s multiple comparisons test: * $p < 0.05$, **** $p < 0.0001$.

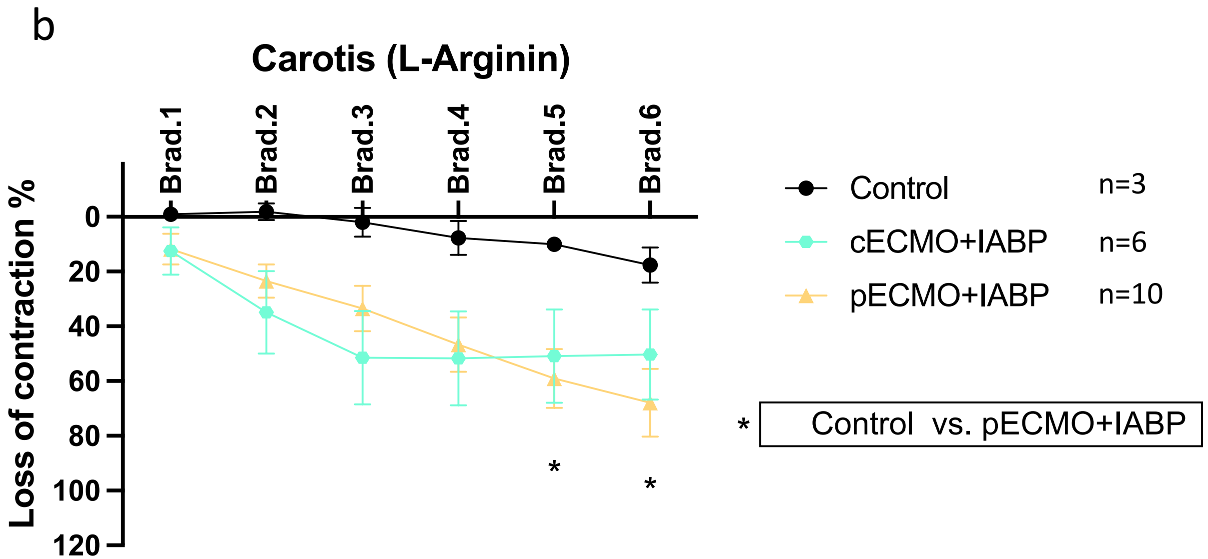

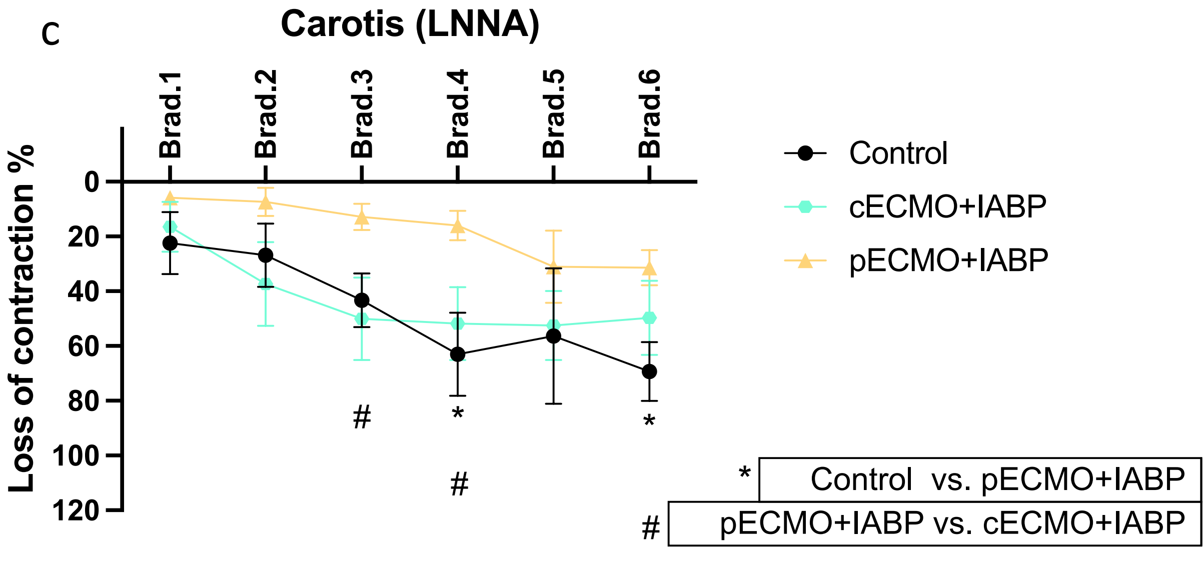

Figure S6

pECMO+IABP vs cECMO+IABP

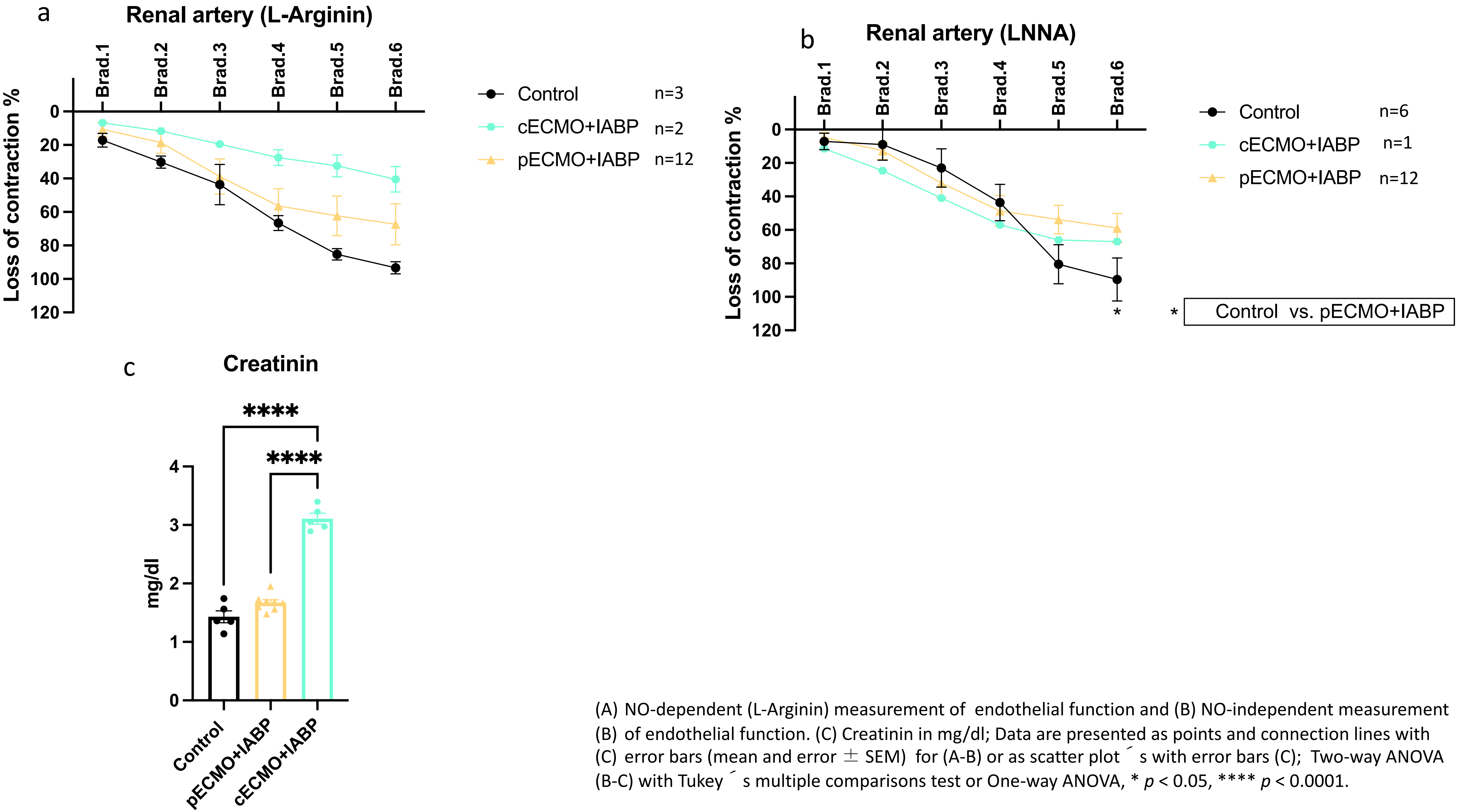

(A) NO-dependent (L-Arginin) measurement of endothelial function and (B) NO-independent measurement
(B) of endothelial function. (C) Creatinin in mg/dl; Data are presented as points and connection lines with
(C) error bars (mean and error ± SEM) for (A-B) or as scatter plot´s with error bars (C); Two-way ANOVA
(B-C) with Tukey´s multiple comparisons test or One-way ANOVA, * $p < 0.05$, **** $p < 0.0001$.
